# Lecture on Belladonna

**Published:** 1885-05

**Authors:** J. T. Kent

**Affiliations:** St. Louis


### LECTURE ON BELLADONNA.

PROFESSOR J. T. KENT, M. D., ST. LOUIS.

CHARACTERISTICS: "*Heat, redness,* and *burning*" are the general characteristics of Bell.; *heat* of the *skin,* but more especially of the *head.* There is burning in the skin wherever there is redness.

The complaints of Bell. are generally active. They come on *suddenly,* continue with more or less violence, and *disappear* as *suddenly* as they came.

It is a deeper acting remedy than Aconite, but not so deep as Sulphur. Its conditions last longer than those of Aconite. When recovery begins, it takes place rapidly.

All the affections of the head are on the right side. The right side of the throat is its common seat of action (mouth, face, and jaws on the left side). Among the acute remedies, Bell. is related to Hyos. and Stram.; both have less heat with their delirium than Bell. Like Bryonia, it has aggravation from motion. Changed condition of mind; delirium, attended with heat of head, redness of face, and burning.

The burning and heat are peculiar. The heat is so marked that when you place your hand on the skin and then remove it, the sensation of the heat remains for some time. The skin is really hot, almost burning. The delirium is attended by a hot, smooth, shining redness of skin.

Intense throbbing of the carotids—a secondary symptom. There is stiffness of the neck and enlargement of cervical glands.

Pains run downward (Silicea has a pain running *up* the back—also Gels.).

HEAD AND MENTAL: Even brain burns—memory may be either impaired or lively. A woman forgets the common duties of her household—forgets in a moment what she was about to do.

Excitement and wild delirium in children. Imagines he sees hideous monsters and insects; wants to jump out of the window. Head is hot and hands and feet cold. Jumps up in his night-clothes, and wants to go out; thinks he is somewhere else. He talks and gesticulates; desires to escape or hide; he is talkative or taciturn; he picks at the bed-clothes; he mutters, and slides down toward the foot of the bed in typhoid (*Mur-acid*, Bapt., Zinc); but Bell. more commonly belongs to this mental condition in acute and active fevers. In women of active minds, who are liable to become delirious in sickness. Bright eyes and shining face in children. Patient sees individuals with black veils rising up. (Opium, Puls., and Stram. have a similar state.) He gets so mad and wild that he barks like a dog. (*Stram.* patient growls and snarls like a dog, but has no great throbbing and redness, though the delirium may be just as active.) She will sit and tear her apron to pieces. In acute mania, with its characteristic head symptoms, Bell. must be the remedy. (If the case tapers off and is not cured, Calcarea will likely follow.) Quarrelsome; breaks into violent fits of laughter; disposed to strike those around; stares in affright at the approach of others.

Will jump up in the night, as if frightened (Sulphur). (Phos. patient imagines something is coming out of a corner.) Anxious face, and is afraid she is going to die.

The Bell. patient is fretful; vexed with himself. (Stramonium has the mental state, but not the intense heat of Bell.) Maniacal delirium in children with this heat. Blood mounts to the head; vertigo.

Bell. has a great many kinds of headache, and a wide range of brain symptoms. With all these headaches there is *throbbing;* they are aggravated by least jar, and by lying down—which causes the blood to flow to the head. Neuralgic headache, periodical headache from 4 to 8 P. M., aggravated by *heat* to head; *cold* gives temporary relief (Ars.). (The application of *cold* makes head *more painful*, Silicea). Headache from heat of the sun. (When a man gets sunstroke, Glonoine.) Every time he goes into the sun he does not perspire; and the more he walks the hotter the skin becomes, when he finally sinks down in collapse, with a great rush of blood to the head. In headache that is brought on by the sun and relieved by Bell., if Bell. does not complete the case give Natrum-mur. (not Calcarea). Vinegar always aggravates the Bell. headache. Cold from having hair cut, or getting head wet (wet scalp). Eyes feel as if starting from their sockets—worse on right side (Spigelia on left side). The Bell. headache is sometimes so severe that patient must stop as he walks (Glon. also) and rest. Pain ameliorated by hard pressure. (Cannabis has a sensation as if the skull were lifted up.) There is a feeling as of a stone on the head; stabbing from one temple to the other; a jolt or jar makes him feel as if he had a weight on the base of the brain.

There may be a soreness to the touch. Tenderness and soreness in rheumatism. (May be mistaken for Arnica. The Arnica patient does not want any one to come near him—he is so sore—fears being touched. Bell. has more of a soreness from a jolt. In Arnica soreness, the bed feels too hard.)

The pains of Bell. come on like lightning, with great violence. The instant they are on, they arrive at their complete intensity. They may last a considerable time, even all night, and finally disappear suddenly.

Head (external) so sensitive that even pressure of the hair causes pain—very often found in congestion of the brain and congestive head troubles.

Eye: Burning, throbbing, dryness of the eye; photophobia, bright sparks before the eyes; objects appear double; deception in vision; halo around the light of a candle; pupils dilated—this will be found in congestion of the brain. You will find patient lying in a stupid condition; throbbing of the carotids; full and bounding pulse; neck drawn back (as in spinal meningitis); shooting in the eyes—*comes* and *goes quickly*. Bell. has inflammation of the caruncula lachrymalis. Bell. eye and head symptoms are made worse by cold drafts of air; yet cold applications will relieve in congestion of the brain. The Bell. patient wants his head covered up (also Silicea and Hepar).

Ears: Much deception in hearing, swelling of the right parotid gland, with stiffness in the cords of the neck, with heat and throbbing.

Face: Bell. is a great face remedy. Face is red and hot, or pale and cold; mottled and swollen. This redness and heat is shining. The measly eruption is not a condition for Bell. (Aconite has a rosy-measly eruption—also Puls. and Euphrasia.)

In scarlet fever, the rash comes out; it shines through, as it were. After the redness of the first stage passes away, there may be coldness of the skin and pallor—in congestion of the brain, and after the rash has been driven in. If this pallor comes on early, Bell. may be the remedy; but if it comes on later, when a plastic deposit is formed, it is (like Aconite) not the remedy. First, fear and anxiety—Aconite; after the localization of the serum in the brain—Bell. (throughout the body—Aconite.) After localization, there is a quiet stupor, delirium; determination of blood to the head; red-hot face. If determination goes beyond this stage, the face is pale, perhaps cold; the eyes sunken; pupils dilated; yet great heat in back part of head, and likely the head is beginning to draw back; pulse, small and weak; now patient has passed into a profound stupor. After exudation, Bell. is no longer the remedy. Now, such remedies as Hellebore, Zincum, and Sulphur may be required. If you get the patient in the first stage, you will not be likely to need Bell., Aconite will do the work. If you have any doubt, and Bell. does not act, exudation has probably taken place. The face tells us all about these conditions. If you have a dilated and fixed pupil, it is a pretty sure sign. There is a peculiar glassy look in the eye, that will be noticed as soon as you enter the room. This condition may be maintained a few days, or may terminate in a few hours. There is a hippocratic countenance that Bell. has no relation to. Here we have to use Opium, Ars., Carbo veg., and Lyc.

In neuralgia the pain is *burning*, violent shooting in the *right* maxillary articulation. (Bell. is to the *right* side what Colocynth is to the *left*). Neuralgia of the upper teeth on the right side, extending back to the temple and ear. Sensation of intense *burning* in the entire face, without redness of the cheeks. Body warm, feet cold. Swelling of the upper lip (Psorinum). *Constant chewing motion* in brain disturbances, though not so prominent as in Stram. and Hellebore. Bry. and Calc. have it in stomach troubles (Bry. also in brain troubles).

Atropia is almost as good as Sulphur in ptyalism. Merc. is the best remedy, but do not forget Bell.

THROAT: Bell. cannot be the remedy if the throat is ulcerated—even if there were throbbing and heat. Bell. should not be given any longer than it would require to select the appropriate remedy. Lach. and Lyc. have all the marked throat features of Bell. It sometimes produces a superficial breaking down, in which the tissues may be bright red, or possibly quite dusky. Enlargement of the glands of the neck on the outside, pain in head, hot face, determination of blood to the head. When soreness commences on the right and goes to the left side, Lyc.; when commencing on the left and going to the right side, Lach. When diphtheria commences in the larynx and ascends, Bromine. The Biniodide of Merc. patient has diphtheria commencing on the left side, left tonsil swollen, fauces dark red, diphtheritic patches, tonsils suppurating, difficult deglutition, worse from empty swallowing. Proto-iodide of Merc. (both have sensation of lump in throat); throat dry; easily detached patches on the inflamed pharynx and fauces, worse on *right* side; fetid discharge. Bell. soreness is bright red, burning, and principally on right side; but if the characteristics are present you might give it for the *left*. When suppuration begins think of Silicea and Baryta carb.

THIRST: Is thirsty; wants lemonade, which does patient good; wants little at a time. All drinks are loathsome. Thirst little and often. (Bell. has this as much as Arsenicum.) Bryonia has a great thirst, but it is for large draughts at long intervals. Ars. patient gets very little relief from drinking; water tastes good, but he soon wants more. Drinking stupefies a young Bell. patient. (*Absence of thirst.*, Apis, *Puls.*, Ipec., and Sabad.)

In congestion of the brain in Bell. we often have vomiting. Bell. is a great liver remedy; region of liver painful and sore to the touch; also acute pain; worse lying on *right* side. *Can tolerate no jar.* In inflammation of the liver, where Bell would be indicated, you need not always look for brain trouble. It comes on *suddenly*, and there is great fever and throbbing pulse. There is that feeling of heat in the skin that I described to you. In painful passage of gall-stone Bell. may relax the common bile-duct. (Berberis is a better remedy for this condition.)

STOOL: Thin, green, mucus, bloody, with tenesmus; chalky, clay-colored lumps. (Several remedies have this stool more characteristic, but this comes on in congestion of the brain; it may occur in dysentery.) The piles of Bell. are so sensitive that the patient has to lie with his nates separated, with throbbing and burning around the anus.

URINE: Paralysis of the sphincter, permitting the urine to escape involuntarily (even when it has existed for a considerable time). Must make great haste to pass his urine. (Have cured this in women with Sepia.) Constant dribbling of urine (where no urine is secreted think of *Ars.* and Colchicum). (Lachesis has suppression of urine, and has the sensation of a ball rolling in the bladder.) Bell. is useful where little girls and children wet the bed. Bell. also has *retention* of urine from paralysis of the bladder.

SEXUAL ORGANS—*female:* Pressure downward, as if all the contents of the abdomen would issue through the vulva. Violent urging and pressure toward the genitals, as if the abdominal viscera would come out—prolapsus uteri. Bell. has more or less

15

throbbing, pressure of blood to the head, sore, dryness, and burning; *better standing* and when *sitting erect* (Sepia, better when *lying down*); Bell. patient is worse when *bent, walking,* and *in the morning.*

*Lilium tig.:* Gets hold of the abdominal walls and tries to lift herself up. A peculiar insane feeling in the head; feels as though she would go crazy.

*Sepia:* Has the bearing-down pains of Bell. and Lilium; also pressure on the vulva. She makes efforts to support herself. The bearing-down is so great that she crosses her limbs to prevent the uterus from protruding. Constipation. Sensation of *a ball in the rectum,* an "all-gone" feeling in the pit of the stomach.

*Nux vomica:* Has a bearing-down, but not that terrible bearing-down of Sepia, Bell., and Lilium, but attended with *irritable* temper and throbbing pain. *Every pain the patient has she has a desire to go to stool.* More severe pains and less dragging down.

*Podophyllum:* Has this dragging down as much as Sepia; seems as if the whole rectum would protrude. Prolapsus of the rectum is complete in Podophyllum.

*Pulsatilla:* The bearing-down pains are not so marked as in Sepia. If patient is a *blonde,* tendency to *weep;* is made better in *open air;* worse in *warm room;* must have doors and windows open.

*Murex:* Has the dragging down of Lilium and of Sepia. She lifts herself up, as in Sepia. Murex has in connection with this severe pains in right and left ovaries; going to the left mammary. Intense sexual desire. (It is very seldom that Sepia has an intense desire for coëtus.) There is a throbbing in the uterus that Sepia does not have. Bell. has a pain in the right ovary. *Clutching pains* anywhere. It is a right-sided remedy in the pelvis. Profuse discharge of bright red blood; the blood that passes is *hot. Hot* blood from nose or any organ. Several remedies have bright red blood. Sometimes blood dark, clotted, and of a bad smell and HOT. In dysmenorrhœa it is important—congestive type, with *heat, burning,* and *dryness* in the uterus. At each menstrual period the blood mounts to the head. Pains come on *violently* and *suddenly.* Chronic inflammation of the uterus with right-sided pains. In girls who are about to menstruate, or who have menstruated and are delayed, Bell. gives relief, but it does not cure. Follow it with Calc-phos. (Painful menstrual disorders in young girls—Calc-phos.) Spasmodic contractions of the uterus and labor-like pains during menses.

PREGNANCY: Lochia is offensive and *hot* to the parts. The os uteris is spasmodically contracted. Irregularity in the contraction of the uterus, with congestion, throbbing of carotids, and delirium. In "milk-leg" Calc. and Sepia are more important remedies, but if the active condition is present, with *beating* of the *veins*, give Bell., following maybe with Calc. Patient appears as if stunned. Renewal of fits at every pain. Retained placenta, with profuse flow of *hot* blood.

In mastitis there is pain and throbbing and redness; streaks radiating from the nipple. (If there is a *hardness*, pain, and throbbing localized in the breast—Phytolacca. It produces a thickening of milk in cows and nodular formations in the udder. Where a knot remains after breast has been injudiciously lanced think of Graphitis.)

EXTREMITIES: Loss of co-ordination of muscles of both upper and lower limbs. Prostration is greatest in the morning. The Bell. patient is always worse after sleep (Cham. and Lach.). In certain affections of the spinal cord, there is an inability to get up after sitting down. Amelioration from *motion*. Aching and throbbing in back, with restlessness and sudden startings. Sleepiness, but cannot sleep (Cham.) Great wakefulness—*Bell.* and *Opium*. Great sleeplessness—Opium and Cham. Sleeps much, yet not refreshed—Bell. and Cham.

After the *active* symptoms of meningitis, and the listless, pale state comes on Bell. is no longer useful, but study Hyos., Veratrum, Lyc., Natrum mur. When the stupor is profound, stertorous breathing and dropping of the lower jaw—Opium. If patient lies stunned, with staring look, in a semi-conscious state, he lies passive, looks not very wild but he is stunned—Arnica. Total unconsciousness—Hellebore. If patient sinks into apathy, as if sensibility was greatly decreased—Hyos. Typhoid Fever: The patient falls asleep in midst of an attempt to answer—Baptisia. In the stupor when he commences to speak to you, but he can't finish his answer, and turns away in disgust—Arnica. After a correct answer he falls into a delirium—Hyos.

SWEAT: Bell. has sweat during sleep (day or night)—a general sweat, *suddenly occurring* and *quickly disappearing*. Sweat during sleep. (Also Puls. Puls. patient is dry and hot during waking state, but as soon as he goes to sleep he will sweat. Sambucus, sweats during *waking state*, drys up when he goes to sleep.)

FEVER: Bell. meets the simple type of remittent fever; *better* in the *morning*, *worse* in the *afternoon*. (Little pimply rash comes out on the face whenever she menstruates, Dulc.).

Renewal of the spasms by contact or glare of light (Stram.).

Rheumatism pains, flying quickly from place to place.

Bell. patient is better in *warm weather*. Especially adapted to plethoric individuals; complaints of scrofulous individuals who take cold easily. Bell. has swelling of the lymphatics. Is adapted to large-headed, brainy children, with blue eyes, blonde hair, and delicate skin; young full-blooded people; high fever and inflammation; threatening convulsions. Bell. is generally indicated in a Calcarea patient, when patient takes cold—there is a determination of blood to the head; after removing the acute state with Bell. follow with Calc. Strong coffee antidotes Belladonna.

[CONDITIONS OF BELLADONNA: Aggravation afternoon and after midnight, from smoking, generally from liquor, being uncovered, swallowing drinks, drinking coffee, eating, after breakfast, warm diet, after sleep, sitting erect, lying on left or painful side, sour things, *least* jar, touching parts even softly, *during* sweat. Amelioration from stooping, sitting bent, lying on right side, *after* sweat, in-doors, bending head backward, on empty stomach, from change of position, while standing.]

